# PGC-1**α**–mediated angiogenesis prevents pulmonary hypertension in mice

**DOI:** 10.1172/jci.insight.162632

**Published:** 2023-09-08

**Authors:** Takayuki Fujiwara, Norifumi Takeda, Hironori Hara, Satoshi Ishii, Genri Numata, Hiroyuki Tokiwa, Manami Katoh, Sonoko Maemura, Takaaki Suzuki, Hiroshi Takiguchi, Tomonobu Yanase, Yoshiaki Kubota, Seitaro Nomura, Masaru Hatano, Kazutaka Ueda, Mutsuo Harada, Haruhiro Toko, Eiki Takimoto, Hiroshi Akazawa, Hiroyuki Morita, Satoshi Nishimura, Issei Komuro

**Affiliations:** 1 Department of Cardiovascular Medicine, The University of Tokyo Hospital, Bunkyo-ku, Tokyo, Japan.; 2 Department of Computational Diagnostic Radiology and Preventive Medicine, The University of Tokyo, Bunkyo-ku, Tokyo, Japan.; 3 Center for Molecular Medicine, Jichi Medical University, Shimotsuke, Tochigi, Japan.; 4 Department of Advanced Translational Research and Medicine in Management of Pulmonary Hypertension, The University of Tokyo, Bunkyo-ku, Tokyo, Japan.; 5 Department of Anatomy, Keio University School of Medicine, Shinjuku-ku, Tokyo, Japan.; 6 Department of Therapeutic Strategy for Heart Failure, and; 7 Department of Advanced Clinical Science and Therapeutics, Graduate School of Medicine, The University of Tokyo, Bunkyo-ku, Tokyo, Japan.

**Keywords:** Angiogenesis, Vascular Biology, Cellular senescence, DNA repair, Endothelial cells

## Abstract

Pulmonary hypertension (PH) is a life-threatening disease characterized by a progressive narrowing of pulmonary arterioles. Although VEGF is highly expressed in lung of patients with PH and in animal PH models, the involvement of angiogenesis remains elusive. To clarify the pathophysiological function of angiogenesis in PH, we compared the angiogenic response in hypoxia (Hx) and SU5416 (a VEGFR2 inhibitor) plus Hx (SuHx) mouse PH models using 3D imaging. The 3D imaging analysis revealed an angiogenic response in the lung of the Hx-PH, but not of the severer SuHx-PH model. Selective VEGFR2 inhibition with cabozantinib plus Hx in mice also suppressed angiogenic response and exacerbated Hx-PH to the same extent as SuHx. Expression of endothelial proliferator-activated receptor γ coactivator 1α (PGC-1α) increased along with angiogenesis in lung of Hx-PH but not SuHx mice. In pulmonary endothelial cell–specific *Ppargc1a*-KO mice, the Hx-induced angiogenesis was suppressed, and PH was exacerbated along with increased oxidative stress, cellular senescence, and DNA damage. By contrast, treatment with baicalin, a flavonoid enhancing PGC-1α activity in endothelial cells, ameliorated Hx-PH with increased *Vegfa* expression and angiogenesis. Pulmonary endothelial PGC-1α–mediated angiogenesis is essential for adaptive responses to Hx and might represent a potential therapeutic target for PH.

## Graphical abstract

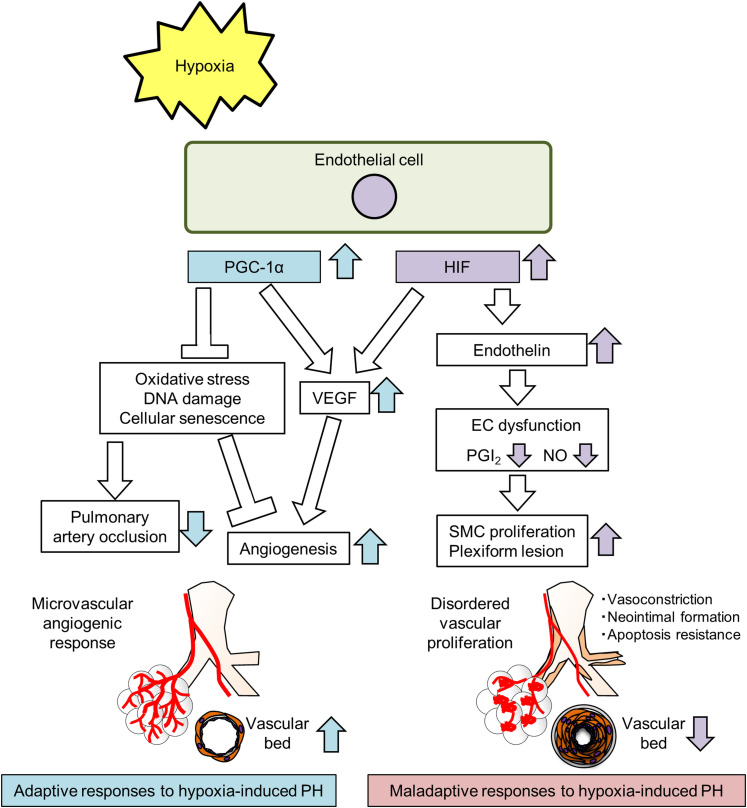

## Introduction

Pulmonary hypertension (PH) is a life-threatening disease characterized by a progressive obliteration of pulmonary arterioles, leading to fatal respiratory and circulatory failure (1). Characteristic histopathological features of PH include proliferation of the smooth muscle cells (SMCs) and endothelial cells (ECs) of the pulmonary arteries (2). Upregulation of VEGF expression was reported in lung tissue from patients with PH (3) and in animal models of PH such as that induced by hypoxia (Hx) (4) and monocrotaline (5). However, the involvement of angiogenesis and VEGF has remained elusive, notably due to inconsistent results. Hemizygosity for either the hypoxia-inducible factor 1 (*Hif1*) (6) or *Hif2* (7) gene, upstream regulators of VEGF, is associated with an attenuation of Hx-PH, but overexpression of adenovirus-mediated lung VEGF-A protected against Hx-PH in rats (8). In addition, SU5416, a VEGFR2-tyrosine kinase inhibitor (TKI), combined with Hx induced severe PH with neointimal occlusive lesions in rats (SuHx) (9), suggesting that VEGF is protective in the context of PH. However, treatment with cabozantinib, a more potent VEGFR2-TKI than SU5416, combined with Hx did not induce severe PH in rats, suggesting that VEGFR inhibition may not be critically involved in the development of severe PH in rats (10).

Recently, we have established a 3D-imaging analysis method for the visualization of murine pulmonary vasculature using clear unobstructed brain- and body-imaging cocktails and computational analysis (CUBIC) (11) combined with multiphoton microscopy. Using this technique, we have successfully generated 3D images of the whole lung at full depth and single-cell resolution in mice, which revealed the previously uncharacterized dynamic of spatial pulmonary vascular remodeling (12) not previously observed using conventional 2D histological examination. Our previous results demonstrated that Hx stimulates the proliferation of ECs and SMCs, which extends to the peripheral lung (12). However, the roles and molecular mechanisms underlying this angiogenic process have remained elusive. The present study examined the significance of the angiogenic response and its roles in the Hx and SuHx mouse models of PH.

## Results

### SMC elongation into the peripheral lung in hypoxic mice is reduced in SU5416-treated hypoxic mice.

**Figure 1:**
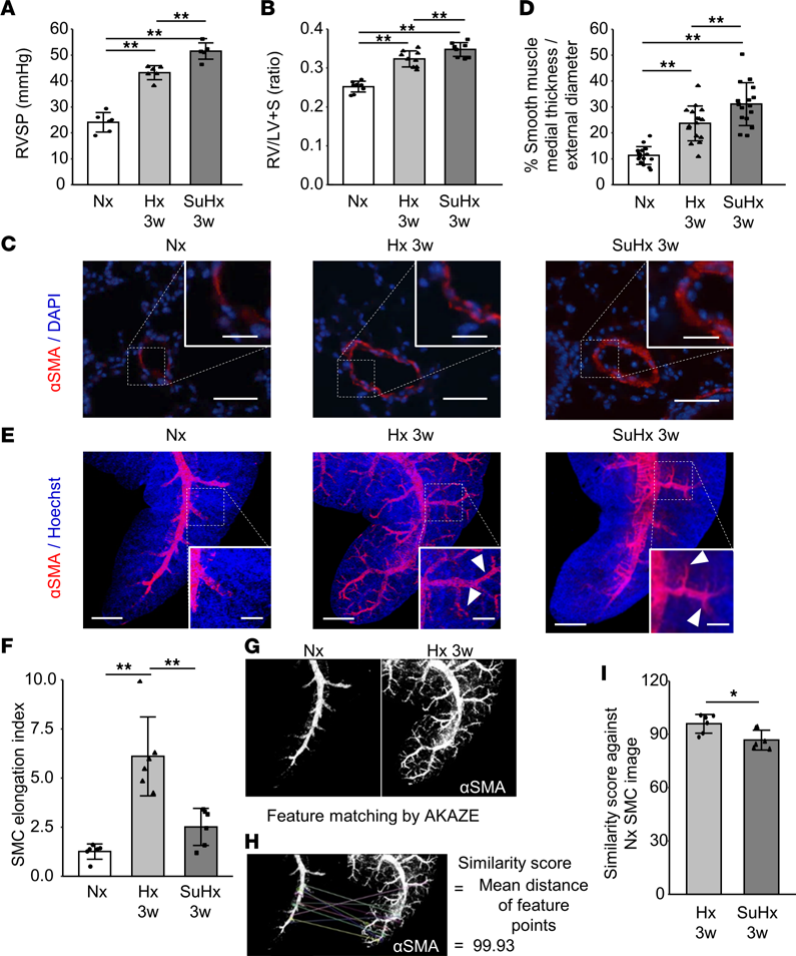
Comparison of PH severity and SMC remodeling between Hx and SuHx mouse models using 2D- and 3D-imaging techniques. (**A**) RVSP in normoxia (Nx), Hx for 3 weeks (Hx 3w), and SU5416 administration under Hx for 3 weeks (SuHx 3w) (*n* = 7 mice/group). (**B**) RVH (*n* = 9 mice/group). (**C**) Representative 2D images of α-SMA–stained arterioles. Scale bar: 50 μm (inset: 20 μm). (**D**) Quantitative analysis of SMC thickness (Nx, *n* = 16 vessels from 4 mice; Hx 3w, *n* = 16 vessels from 3 mice; SuHx 3w, *n* = 16 vessels from 4 mice). (**E**) Representative 3D images of α-SMA–stained arteries. Arrowheads indicate SMC elongation. Scale bar: 500 μm (inset: 200 μm). (**F**) SMC elongation index (*n* = 6 mice/group). (**G**) Automated quantification using the AKAZE feature-matching algorithm. The feature points of the images were automatically extracted from 2 images. (**H**) The similarity degree of images was calculated by mean distance between the matched feature points. (**I**) Similarity score against Nx SMC image (*n* = 6 mice/group). Images for all the comparisons are provided in Supplemental Figure 1. Data are representative of 3 independent experiments. Statistical significance for comparison between means was evaluated using a 1-way ANOVA, followed by Tukey’s multiple-comparison post hoc tests. *P < 0.05, **P < 0.01.

Both Hx and SuHx mice exhibited elevated right ventricular systolic pressure (RVSP) and right ventricular hypertrophy (RVH). Compared with Hx mice, RVSP and RVH were significantly greater in SuHx mice (Figure 1, A and B). Conventional 2D histopathological analysis revealed increased α–smooth muscle actin (α-SMA) staining in the medial area of the arterioles in both PH models, and the vascular SMC layer was significantly thicker in SuHx mice compared with Hx mice (Figure 1, C and D). The 3D imaging of α-SMA–stained SMCs further indicated an elongation of vessels into the peripheral lung tissue of Hx mice, but not SuHx mice, which was quantitatively evaluated by SMC elongation index (12) (Figure 1, E and F, and Supplemental Videos 1–3; supplemental material available online with this article; https://doi.org/10.1172/jci.insight.162632DS1). Feature-matching analysis using the AKAZE feature detector and descriptor (13) showed that the similarity score of 3D-reconstructed SMC images of SuHx against normoxia was significantly lower than that of Hx mice against normoxia (Figure 1, G–I, and Supplemental Figure 1). This finding also suggests that 3D SMC remodeling is less marked in SuHx mice than in Hx mice.

### Neovessel formation after hypoxic exposure is associated with an upregulation of proliferator-activated receptor γ coactivator 1α in the pulmonary endothelium.

**Figure 2:**
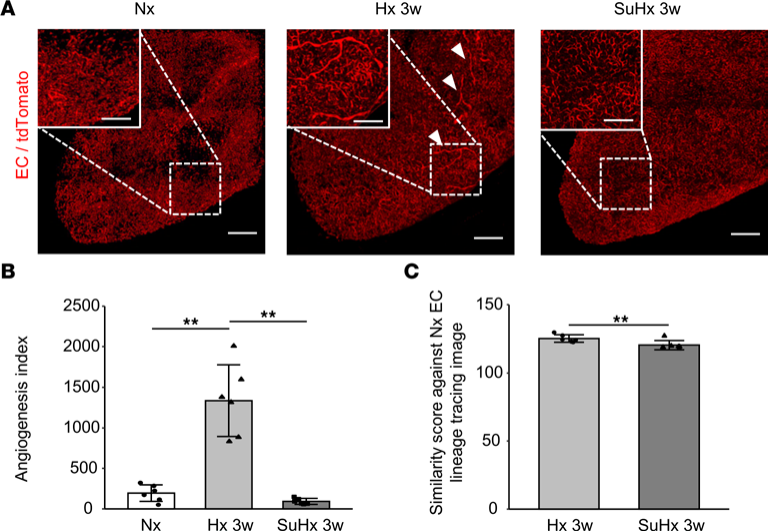
3D EC lineage-tracing visualized the angiogenic response in Hx-PH mouse model. (**A**–**C**) 3D EC lineage-tracing experiment in postcaval lobe using *VE-cadherin-CreER^T2^* *Rosa26-lsl-tdTomato* mice. (**A**) Sprouting and elongation of the tdTomato-labeled preexisting ECs was evident in Hx 3w PH mice (arrowheads), whereas SuHx PH mice had less angiogenesis. Scale bar: 200 μm (inset: 100 μm). (**B**) Angiogenesis index (*n* = 6 mice/group). (**C**) Similarity score against normoxia (Nx) EC lineage-tracing image (*n* = 6 mice/group). Images for all the comparisons are provided in Supplemental Figure 2. Statistical significance for comparison between means was evaluated using a Student’s *t* test or a 1-way ANOVA followed by Tukey’s multiple-comparison post hoc tests. **P < 0.01. Hx 3w, Hx for 3 weeks.

We sought to characterize SMC remodeling patterns and to assess the involvement of angiogenesis with proliferation of existing SMCs surrounding EC sprouts because SMCs were reported to mediate the muscularization of previously nonmuscularized arterioles in the context of PH (14). We performed 3D EC lineage-tracing experiments by crossing *Rosa26-lsl-tdTomato* reporter mice with mice carrying a *VE-cadherin-CreER^T2^*–inducible endothelial Cre driver (12). Tamoxifen-induced labeling of preexisting ECs using tdTomato fluorescence revealed marked EC sprouting and elongation during PH development in Hx mice, which was evaluated with the angiogenesis index (12). This angiogenic response was reduced in SuHx mice (Figure 2, A and B, and Supplemental Videos 4–6). The similarity score of EC lineage-tracing 3D-reconstructed images further indicated that neovessel formation was dampened in SuHx mice compared with Hx mice (Figure 2C and Supplemental Figure 2).

**Figure 3:**
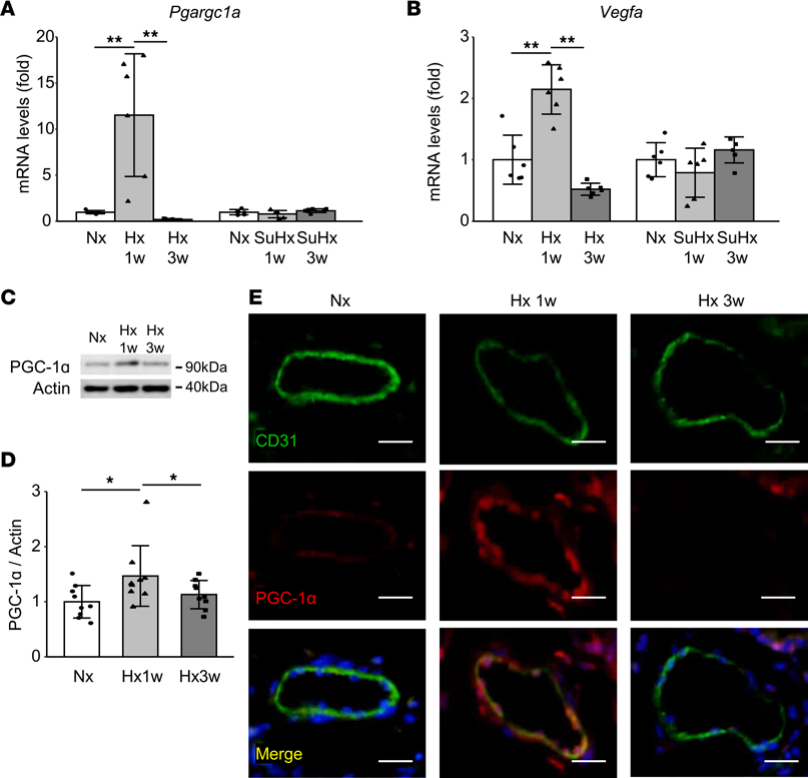
Upregulation of endothelial PGC-1a expression in the early phase of Hx-PH. (**A** and **B**) qRT-PCR analysis of *Ppargc1a* (**A**) and *Vegfa* (**B**) gene expression in the lung at the indicated time points in Hx and SuHx mice (*n* = 6 mice/group). (**C** and **D**) Western blot analysis of PGC-1α protein in the lung of normoxic (Nx), Hx 1w, and Hx 3w mice. One representative blot (**C**) and the quantified PGC-1α levels (**D**) are presented (*n* = 9 mice/group). (**E**) Representative images of arterioles from Nx, Hx 1w, and Hx 3w mice, stained with CD31 and PGC-1α Abs. Nuclei (blue) were labeled with DAPI. Scale bar: 20 μm. Data are representative of 3 independent experiments. Statistical significance for comparison between means was evaluated using a 1-way ANOVA followed by Tukey’s multiple-comparison post hoc tests. *P < 0.05, **P < 0.01. Hx 1w (3w), Hx for 1 week (3 weeks).

ECs play a key role in angiogenesis. At the molecular level, the HIF-independent angiogenesis regulator proliferator-activated receptor γ coactivator 1α (PGC-1α) and its responsive genes play crucial roles in hypoxic-ischemic conditions (15, 16), and PGC-1α was found to be downregulated in patients with advanced PH and in animal models of the pathology (17); the PGC-1α activator metformin ameliorates Hx-PH (18). We assumed, therefore, that PGC-1α exerts a crucial influence on the angiogenic process and disease progression in PH, similar to the angiogenic response and blood flow recovery in the hind limb ischemia model (15). Although the expression of *Ppargc1a* encoding PGC-1α was found to be unaltered 3 weeks after exposure to hypoxic conditions, as in normoxia mice, expression of *Ppargc1a* and *Vegfa* was increased in Hx, but not SuHx, at 1 week after exposure (Figure 3, A and B). In addition, we found that the levels of PGC-1α protein were increased at 1 week but downregulated at 3 weeks after exposure (Figure 3, C and D). Immunostaining confirmed this temporal increase of PGC-1α in the endothelium of arterioles during the early phase of pathology in Hx-PH mice (Figure 3E).

**Figure 4:**
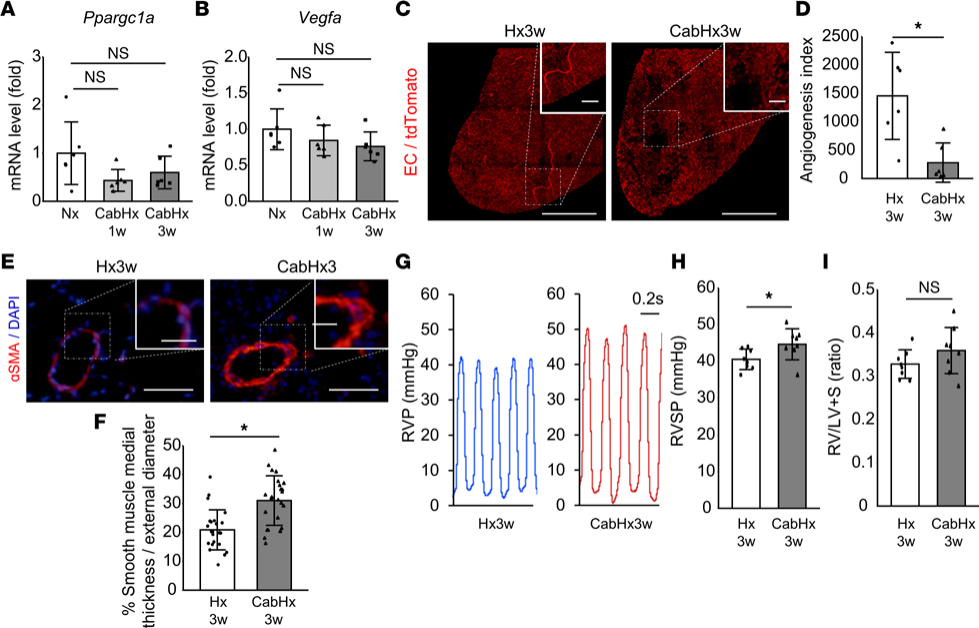
Cabozantinib, a highly potent VEGFR2 inhibitor, exacerbated Hx-PH with suppression of *Ppargc1a* expression and angiogenic response. Hx mice were treated with cabozantinib (CabHx) or vehicle. (**A** and **B**) Relative expression of *Ppargc1a* (**A**) and *Vegfa* (**B**) measured 1 week and 3 weeks after hypoxic exposure (*n* = 6 mice/group). (**C** and **D**) 3D EC lineage-tracing experiment. (**C**) Representative images showing reduced angiogenic responses induced by cabozantinib. Scale bar: 500 μm (inset: 100 μm). (**D**) Angiogenesis index (*n* = 6 mice/group). (**E**) Representative 2D images showing α-SMA–stained arterioles. Scale bar: 50 μm (inset: 20 μm). (**F**) Quantification of SMC thickness (*n* = 24 vessels from 4 mice/group). (**G**) Representative RVP data. (**H**) RVSP (*n* = 8 mice/group). (**I**) RVH (*n* = 8 mice/group). Data are representative of 3 independent experiments. Statistical significance for comparison between means was evaluated using a Student’s *t* test. *P < 0.05. S, septum.

To verify the contribution of VEGFR2 inhibition to the exacerbation of Hx-PH with suppression of *Ppargc1a* and *Vegfa* expression, and to angiogenic response, we administered cabozantinib, a highly potent VEGFR2 inhibitor, to Hx-PH mice (CabHx). After exposure to hypoxic conditions with the administration of cabozantinib, the expression of *Ppargc1a* and *Vegfa* was not upregulated (Figure 4, A and B), and the angiogenic response was reduced compared with Hx-PH (Figure 4, C and D, and Supplemental Videos 7 and 8) and SuHx mice. In addition, vascular SMC thickness (Figure 4, E and F) and RVSP (Figure 4, G and H) increased in CabHx mice compared with Hx mice, whereas RVH was not enhanced (Figure 4I). These results suggest that VEGFR2 inhibition exacerbates PH in mice with suppression of *Ppargc1a* and *Vegfa* and reduction in angiogenic response.

### Pulmonary endothelial cell–specific Ppargc1α deficiency exacerbates Hx-PH with decreased Vegfa expression and lack of angiogenic response after hypoxic exposure.

**Figure 5:**
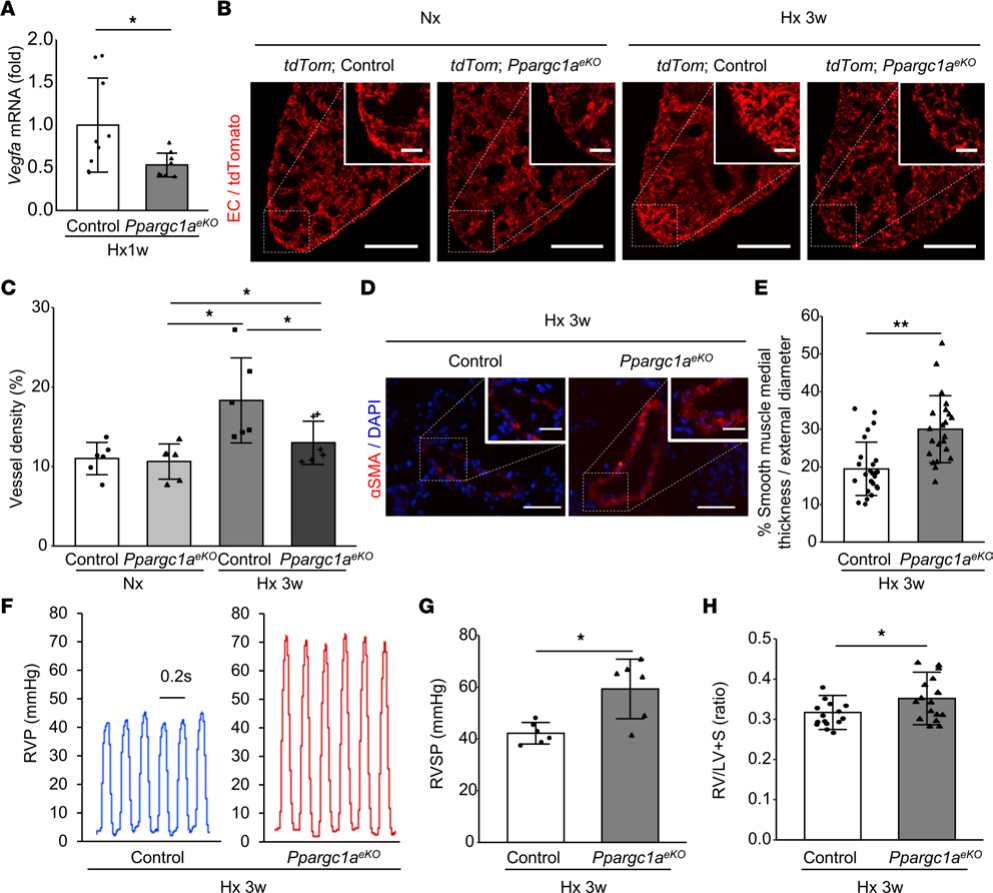
Decreased angiogenic response and deterioration of PH after Hx in EC-specific *Ppargc1a*-deficient mice. (**A**) Relative expression of *Vegfa* in the lung (*n* = 9 mice/group). Pulmonary *Ppargc1*α*^eKO^* and control (*Ppargc1*α*^fl/fl^*) mice were exposed to Hx for 1 week (Hx 1w). (**B** and **C**) Pulmonary ECs of *Ppargc1*α*^eKO^* and control (*Ppargc1*α*^+/+^*) mice were labeled with tdTomato fluorescence by generating *tdTom* *Ppargc1*α*^eKO^* and *tdTom* control mice, respectively. The mice were exposed to normoxic (Nx) or Hx for 3 weeks (Hx 3w). (**B**) Representative multiphoton images of the lung ECs. Scale bar: 500 μm (inset: 200 μm). (**C**) Quantification of EC volume density (*n* = 6 mice/group). (**D**–**H**) *Ppargc1*α*^eKO^* and control (*Ppargc1*α*^fl/fl^*) mice were exposed to Hx for 3 weeks. (**D**) Representative 2D images showing α-SMA–stained section of distal arteries. Scale bar: 50 μm (inset: 20 μm). (**E**) Quantitative analysis of SMC thickness in distal arteries (control, *n* = 26 vessels in 4 mice; *Ppargc1*α*^eKO^*, *n* = 22 vessels in 4 mice). (**F**) Representative RVP data. (**G**) RVSP (*n* = 6 mice/group). (**H**) RVH (control, *n* = 15 mice; *Ppargc1*α*^eKO^*, *n* = 17 mice). Data are representative of 3 independent experiments. Statistical significance for comparison between means was evaluated using a Student’s *t* test or a 1-way ANOVA, followed by Tukey’s multiple-comparison post hoc tests. *P < 0.05, **P < 0.01. S, septum.

To further examine the role of PGC-1α in the endothelium during PH development in Hx mice, we generated endothelial specific *Ppargc1a*-deficient mice (*L1-Cre* *Ppargc1a^fl/fl^* mice; hereafter, *Ppargc1a^eKO^*) by mating mice expressing *loxP*-flanked alleles of *Ppargc1a* with mice producing Cre recombinase predominantly in the pulmonary ECs under control of the *Alk1* promoter (*L1-Cre*) (19). After a week of hypoxic exposure, *Ppargc1a^eKO^* mice exhibited decreased expression of *Vegfa* in the early phase of Hx-PH pathology compared with control mice (*Ppargc1a^fl/fl^*) (Figure 5A).

Labeling of pulmonary ECs with tdTomato fluorescence in *Ppargc1a^eKO^* and control (*Ppargc1a^+/+^*) mice was obtained by generating *L1-Cre Rosa26-lsl-tdTomato Ppargc1*α*^fl/fl^* (*tdTom* *Ppargc1a^eKO^*) and *L1-Cre Rosa26-lsl-tdTomato Ppargc1a^+/+^* (*tdTom* control) mice, respectively. EC volume was evaluated by calculating vessel density, which revealed no difference in microvascular density between the *tdTom* *Ppargc1a^eKO^* mice and *tdTom* control mice at baseline in normoxia (Figure 5, B and C). After 3 weeks of hypoxic exposure, microvascular density was increased in *tdTom* control mice, contrary to *tdTom* *Ppargc1a^eKO^* mice (Figure 5, B and C). These results suggest that pulmonary endothelial PGC-1α plays an important role in the angiogenic response, notably by stimulating *Vegfa* expression after hypoxic exposure.

Our investigation of *Ppargc1a^eKO^* mice revealed that pulmonary endothelial PGC-1α deficiency results in increased SMC thickness (Figure 5, D and E) accompanied by enhanced RVSP and RVH after 3 weeks of hypoxic exposure (Figure 5, F–H).

### Endothelial PGC-1α dysregulation enhances vascular oxidative stress, cellular senescence, and DNA damage after hypoxic exposure.

**Figure 6:**
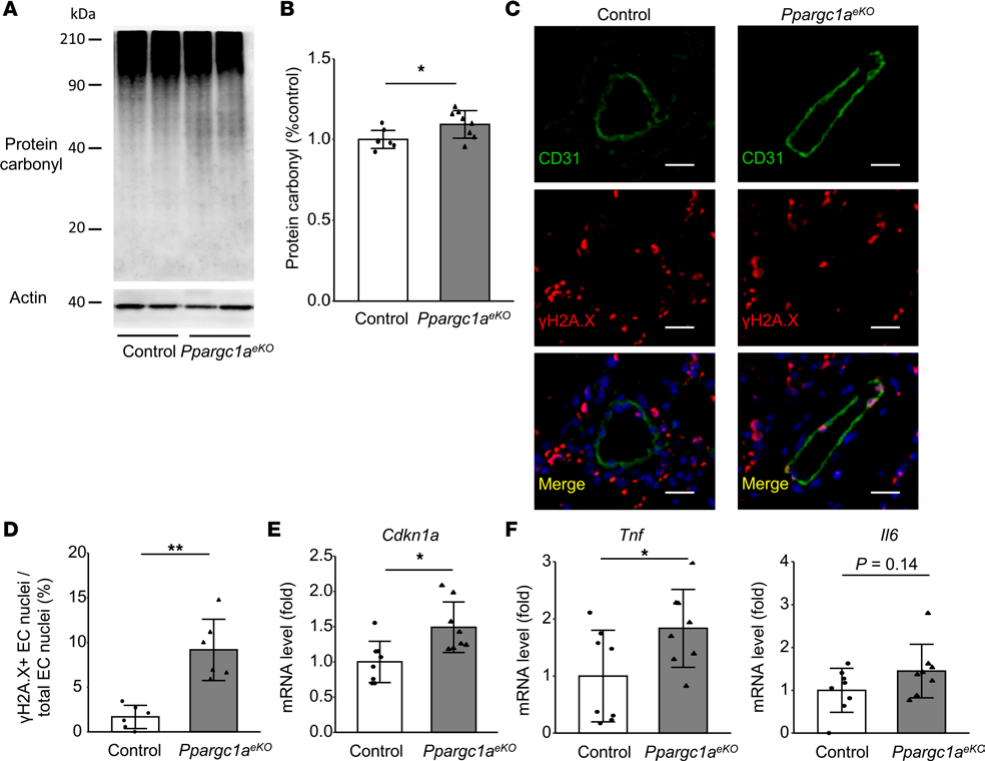
Increased cellular senescence after Hx in pulmonary EC-specific *Ppargc1a*-deficient mice. *Ppargc1*α*^eKO^* and control (*Ppargc1*α*^fl/fl^*) mice were exposed to Hx for 3 weeks (Hx 3w). (**A** and **B**) Western blot analysis of protein carbonyl in the lung. Representative blots (**A**) and quantified protein carbonyl levels (**B**) are presented (*n* = 8 mice/group). (**C** and **D**) γH2A.X expression. (**C**) Representative images showing distal pulmonary arteries stained with CD31 and γH2A.X Abs. Nuclei (blue) were labeled with DAPI. Scale bar: 20 μm. (**D**) The proportion of γH2A.X^+^ nuclei in ECs forming arterioles (*n* = 6 mice/group). (**E**) Relative expression of *Cdkn1a* (*n* = 8 mice/group). (**F**) Relative expression of SASP markers of *Tnf* and *Il6* (*n* = 8 mice/group). Data are representative of 3 independent experiments. Statistical significance was evaluated using a Student’s *t* test for comparison between means and using a Pearson’s χ^2^ test for comparison of the proportion of γH2A.X^+^ nuclei in ECs. *P < 0.05, **P < 0.01.

We next examined the effect of inhibiting PGC-1α signaling on endothelial dysfunction during Hx-PH development. The total protein carbonyl, an oxidative stress marker, was increased in the lungs of *Ppargc1a^eKO^* mice compared with control mice (Figure 6, A and B). Immunostaining for γH2A.X revealed increased DNA damage in ECs (Figure 6, C and D). The expression of *Cdkn1a*, a senescence marker encoding p21 (Figure 6E), and the expression of *Tnf*, considered a senescence-associated secretory phenotype (SASP) marker (Figure 6F), were significantly increased in *Ppargc1a^eKO^* mice compared with control mice. These results suggest that the dysregulation of pulmonary endothelial PGC-1α impairs angiogenesis, exacerbates the effects of oxidative stress, and increases cellular senescence and DNA damage. Together, these effects may contribute to driving the progression of Hx-PH.

### Activation of PGC-1α by baicalin enhances the angiogenic response and ameliorates the development of PH after hypoxic exposure.

**Figure 7:**
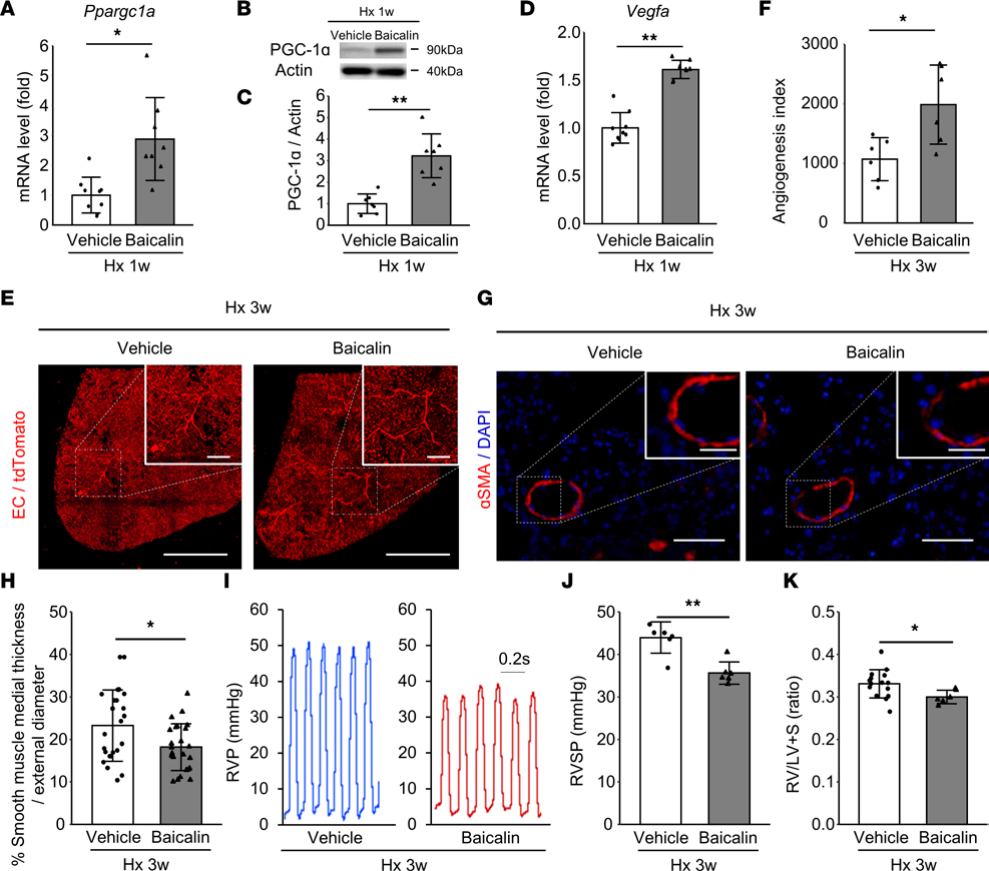
Increased angiogenic response and ameliorated PH upon PGC-1α activation by baicalin in the Hx-PH mouse model. Mice were treated with baicalin (150 mg/kg/d) or vehicle during 3 weeks of hypoxic conditions (Hx 3w). (**A**) Relative expression of *Ppargc1a* measured 1 week after hypoxic exposure (vehicle, *n* = 9 mice; baicalin, *n* = 8 mice). (**B** and **C**) Western blot analysis of PGC-1α protein in the lung. One representative blot (**B**) and quantified PGC-1α levels (**C**) are presented (*n* = 7 mice/group). (**D**) Relative expression of *Vegfa* 1 week after hypoxic exposure (vehicle, *n* = 9 mice; baicalin, *n* = 8 mice). (**E** and **F**) 3D EC lineage-tracing experiment. (**E**) Representative images showing increased angiogenic responses induced by baicalin. Scale bar: 500 μm (inset: 100 μm). (**F**) Angiogenesis index (*n* = 6 mice/group). (**G**) Representative 2D images showing α-SMA–stained arterioles. Scale bar: 50 μm (inset: 20 μm). (**H**) Quantification of SMC thickness (*n* = 24 vessels from 4 mice per group). (**I**) Representative RVP data. (**J**) RVSP (*n* = 6 mice/group). (**K**) RVH (vehicle, *n* = 15 mice; baicalin, *n* = 6 mice). Data are representative of 3 independent experiments. Statistical significance for comparison between means was evaluated using a Student’s *t* test. *P < 0.05, **P < 0.01.

**Figure 8:**
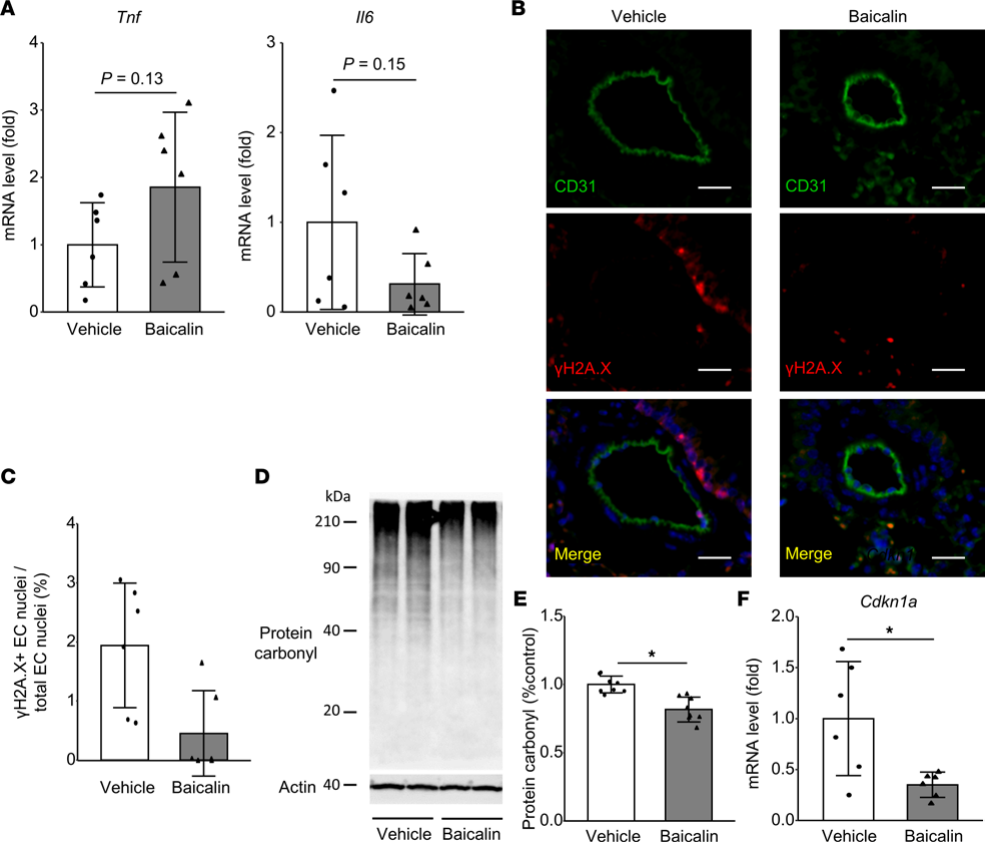
Suppressed Hx-induced cellular senescence of ECs upon PGC-1α activation by baicalin. Mice were treated with baicalin (150 mg/kg/d) or vehicle during 3 weeks of hypoxic conditions (Hx 3w). (**A**) Relative expression of SASP markers (*n* = 6 mice/group). (**B**) Representative images showing distal pulmonary arteries stained with CD31 and γH2A.X Abs. Nuclei (blue) were labeled with DAPI. Scale bar: 20 μm. (**C**) Proportion of γH2A.X^+^ nuclei in ECs of distal pulmonary arteries (*n* = 6 mice per group). (**D** and **E**) Western blot analysis of protein carbonyl in the lung. Representative blots (**D**) and quantified protein carbonyl levels (**E**) are presented (*n* = 8 mice/group). (**F**) Relative expression of *Cdkn1a* in the lung (*n* = 6 mice/group). Data are representative of 3 independent experiments. Statistical significance was evaluated using a Student’s *t* test for comparison between means and using a Pearson’s χ^2^ test for comparison of the proportion of γH2A.X^+^ nuclei in ECs. *P < 0.05.

Given the angiogenic response and the simultaneous temporal increase of PGC-1α in Hx-PH mice, not observed in the severer SuHx model, angiogenesis was hypothesized to exert a key role in the structural and functional adaptation of Hx mice to hypoxic conditions. To test this possibility, hypoxic mice received an i.p. injection of baicalin, a flavonoid derived from the roots of *Scutellaria baicalensis*, which increases *Vegfa* expression and angiogenesis by activating the ERRα/PGC-1α complex in HUVECs (20). Baicalin treatment (150 mg/kg/d) increased the expression levels of PGC-1α (Figure 7, A–C) and *Vegfa* (Figure 7D) in lung tissue 1 week after hypoxic exposure. Our 3D EC lineage-tracing data indicated that baicalin significantly enhances neovessel formation in *VE-cadherin-CreER^T2^ Rosa26-lsl-tdTomato* mice (Figure 7, E and F, and Supplemental Videos 9 and 10). The increased SMC thickness, RVSP, and RVH were significantly ameliorated in Hx-PH mice upon baicalin treatment (Figure 7, G–K). Baicalin treatment did not significantly affect the expression of SASP markers (Figure 8A) but decreased oxidative stress markers, *Cdkn1a* expression, and the number of ECs with γH2A.X in Hx-PH mice (Figure 8, B–F).

To examine whether the beneficial angiogenic effect of baicalin was mediated specifically through PGC-1α activation in ECs, we administered baicalin to *Ppargc1a^eKO^* mice in hypoxic conditions because baicalin could inhibit SMC proliferation in vitro (21). Baicalin treatment did not enhance PGC-1α protein (Supplemental Figure 3, A–C), *Vegfa* gene expression (Supplemental Figure 3D), or angiogenic response (Supplemental Figure 3, F and G), and could not attenuate Hx-PH model in *Ppargc1a^eKO^* mice (Supplemental Figure 3, G–K).

Finally, we treated SuHx mice with baicalin to determine whether other protective pathways might be induced that might be different than just induction of VEGF, because PGC-1α has potentially favorable effects on vascular remodeling in PH (17, 18). Although baicalin treatment enhanced the PGC-1α protein expression (Supplemental Figure 4, A–C), the *Vegfa* gene was not upregulated (Supplemental Figure 4D) and the angiogenic response was not induced (Supplemental Figure 4, E and F, and Supplemental Videos 11 and 12). The increased SMC thickness, RVSP, and RVH were not ameliorated in SuHx mice by baicalin treatment (Supplemental Figure 4, G–K). These findings suggest that baicalin ameliorated Hx-PH through a PGC-1α–VEGF-mediated angiogenic response.

## Discussion

Recent developments in 3D imaging combined with tissue-clearing techniques have greatly increased the understanding of human diseases in various fields of medicine, including neurology (11) and nephrology (22). In the present study, we aimed to evaluate pulmonary vascular remodeling in the Hx and SuHx mouse models using our novel 3D-imaging system to explore the underlying mechanisms of PH pathology varying in severity.

An angiogenic response extending to the peripheral lung tissue was markedly observed in Hx, whereas the severer SuHx model showed less angiogenesis, and angiogenesis was associated with a temporal increase in PGC-1α expression. The mouse CabHx model also suppressed angiogenic response and exacerbated PH compared with the Hx-PH as well as SuHx models. The *Ppargc1a^eKO^* mice were devoid of an angiogenic response and exhibited a deterioration of Hx-PH with increased oxidative stress, cellular senescence, and DNA damage. By contrast, PGC-1α activation led to increased angiogenesis and an overall amelioration of Hx-PH phenotypes. These results suggested that pulmonary endothelial PGC-1α–mediated angiogenesis is essential for adaptive responses to Hx and might represent a potential therapeutic target for PH.

The number of patients with PH is increasing, and combination therapy with pulmonary vasodilators, including prostacyclin pathway agonists, endothelin receptor antagonists, and nitric oxide–cyclic guanosine monophosphate enhancers, has been shown to improve prognosis and quality of life (23). Numerous studies have measured the expression of many related genes and proteins in the context of human PH, including VEGF (23). However, the mechanisms underlying PH development have remained elusive, and targeted therapies specific to PH are still lacking.

We recently developed a novel 3D-imaging system enabling the visualization of the murine pulmonary vasculature. The use of this system revealed a significant proliferation of ECs, together with SMCs, extending to the peripheral lung tissue in the Hx-PH mouse model (12). This response was not detected using conventional 2D histological examination. With this novel 3D-imaging system, the EC proliferation also was found to be more obvious in Hx-PH than in the severer SuHx model of PH. The angiogenic response patterns reflected the expression of the angiogenesis-related genes *Vegfa* and *Ppargc1a* in the acute phase after hypoxic exposure. This newly identified angiogenic response appears to be essential for the adaptation to acute hypoxic exposure and maintenance of EC function but was suppressed after treatment with SU5416, a VEGFR2 TKI. It has been shown that cabozantinib, which is a more potent VEGFR2 TKI, could not induce severe PH in hypoxic rats and SU5416 might exert a pathogenic effect via combined VEGFR2 and BMPR2 signaling inhibition (10) and/or activation of aryl hydrocarbon receptor signaling (24) under hypoxic conditions. However, in the present study, administration of cabozantinib to hypoxic mice exacerbated Hx-PH with suppression of *Ppargc1a* and *Vegfa* expression as well as SU5416. These conflicting results should be due to the difference in response to VEGFR TKI between rats and mice, and further investigations are required to confirm that VEGFR2 inhibition itself induces exacerbation of Hx-PH in mice.

Previous reports have indicated that PGC-1α–responsive genes contribute to metabolic processes by regulating the activity of many transcription factors related to mitochondrial biogenesis, inflammation, and metabolic signaling (25). Among them, HIF-independent VEGF signaling is a key pathway. *Vegf* gene expression is controlled by PGC-1α via the orphan nuclear receptor ERRα, which plays a crucial role in blood flow recovery after hind limb ischemia (15). The present study demonstrates that PGC-1α is upregulated after hypoxic exposure, which leads to an upregulation of *Vegf* expression and increased angiogenesis in Hx-PH mice.

PGC-1α also plays important roles in suppressing mitochondrial oxidative stress and dysfunction in vascular cells, which could suppress cellular senescence. Underlying mechanisms include induction of mitochondrial antioxidant proteins including MnSOD, catalase, Prx5, Prx3, UCP2, TRXR2, and TRX2 (26); prevention of angiotensin II–mediated JNK activation (27); and amelioration of angiotensin II–induced vascular senescence by enabling forkhead box protein O1 (FoxO1)-dependent sirtuin 1 (SIRT1) transcription (28). In humans as well as in various rodent models of PH (29, 30), pulmonary endothelial senescence is considered a cause of inflammation-associated pulmonary endothelial remodeling and dysfunction. Furthermore, SASP of senescent pulmonary ECs was shown to be associated with increased proliferation in cultured pulmonary vascular SMCs (30). Accordingly, we hypothesized that pulmonary endothelial PGC-1α is a key regulator of cellular senescence that may prevent the progression of PH. We investigated the role of pulmonary endothelial PGC-1α in cellular senescence and demonstrated that pulmonary endothelial PGC-1α attenuated Hx-induced oxidative stress, cellular senescence, and DNA damage. Upregulation of pulmonary endothelial PGC-1α might, therefore, promote adaptive angiogenic responses by increasing the expression of VEGF as well as reducing endothelial cellular senescence, DNA damage response, and oxidative stress. Taken together, these mechanisms might suppress the progression of Hx-PH pathology.

PGC-1α is regulated at both transcriptional and posttranscriptional levels. The transcriptional regulation of PGC-1α is mainly mediated by cyclic AMP response element–binding protein (CREB) (31), myocyte enhancer factor 2 (MEF2) (32), and FoxO1 (33) transcription factors. SIRT1 can alter PGC-1α activity by changing its acetylation status (34), and AMPK regulates both the acetylation status and expression of PGC-1α (34, 35). In patients and animals with chronic advanced PH, decreased expression and activity of AMPK (36) and PGC-1α (17) have been reported. Consistently, mice deficient in SMC-specific *Creb* (37) or *Foxo1* (38), EC-specific *Mef2c* (39), *Ampk* (36), or inducible systemic *Sirt1* (40) exhibited deterioration of PH. Additionally, several studies have shown that increased expression and/or activity of PGC-1α–related factors can prevent the progression of PH. The activation of FoxO (41), MEF2 (39), or AMPK (36) attenuates the progression of PH in mouse models. Our findings in Hx-PH mice support these previous results, indicating that therapies aimed at increasing the activation of PGC-1α could attenuate or prevent pulmonary vascular remodeling in PH.

Overall, our results demonstrate that pulmonary endothelial PGC-1α mediates EC protection and angiogenesis in hypoxic mice. These mechanisms might represent a potential avenue for the development of targeted therapies for the prevention and treatment of PH.

## Methods

### Reagents and solutions.

Paraformaldehyde (PFA) (CAS 30525-89-4), urea (CAS 57-13-6), sucrose (CAS 57-50-1), and 2,2’,2’’-nitrilotriethanol (CAS 102-71-6) were purchased from Wako Pure Chemical Industries, Ltd. Polyethylene glycol mono-*p*-isooctylphenyl ether (Triton X-100; CAS 9002-93-1) was purchased from Nacalai Tesque. N,N,N’,N’-tetrakis (2-hydroxypropyl) ethylenediamine (CAS 102-60-3) was purchased from Tokyo Chemical Industry Co., Ltd.

### Animals.

Tg(*Alk1*-cre)-L1 (*L1-Cre*) mice on an FVB background (19) and Tg(*Cdh5*-cre*/ERT2*) (*VE-cadherin-CreER^T2^*) mice on a C57BL/6 background (42) have been previously described. *Rosa26*-*lsl-tdTomato* mice on a C57BL/6 background (43) and *Ppargc1*α*^fl/fl^* mice on a 129 background (44) were purchased from Jackson Laboratory. C57BL/6J mice were purchased from CLEA Japan, Inc. Tg(*Alk1*-cre)-L1 (*L1-Cre*) and *Ppargc1*α*^fl/fl^* mice were backcrossed onto C57BL/6J background for 10 generations before producing experimental animals. Male mice were used for the experiments. All personnel involved in data collection and analysis were masked to the treatment allocation.

All mice were housed on a 12-hour light/12-hour dark cycle at 24°C ± 1°C and were provided with standard mouse food and water ad libitum. The mice either were housed under standard normoxic conditions or were continuously housed in a hypoxic chamber (8.5% O_2_) (12) at sea-level atmospheric pressure at 8 weeks of age for up to 1 week or 3 weeks, except for a 5-minute interval once a week when the chamber was cleaned or when a drug was percutaneously administered, and once a day when a drug was administered i.p. The hypoxic gas mixture was continuously delivered from a nitrogen gas generator MNT-0.4SI (KOFLOC) to the chamber at a flow rate of approximately 1 L/min.

### Drug administration.

SU5416 (CAS 204005-46-9) was purchased from Bio-Techne. SU5416 was suspended using sonication in a mixture of 0.5% carboxymethylcellulose sodium (CAS 9004-32-4), 0.9% sodium chloride (CAS 7647-14-5), 0.4% polysorbate 80 (CAS 9005-65-6), and 0.9% benzyl alcohol (CAS 100-51-6) in deionized water (all from Wako Pure Chemical Industries, Ltd). SuHx mice were injected once weekly with SU5416 at 20 mg/kg BW during hypoxic exposure (45). Cabozantinib was purchased from LC Laboratories and suspended using sonication in a mixture of 0.5% carboxymethylcellulose sodium, 0.9% sodium chloride, 0.4% polysorbate 80, and 0.9% benzyl alcohol in deionized water. CabHx mice were injected once weekly with cabozantinib at 20 mg/kg BW during hypoxic exposure (10). Baicalin (CAS 21967-41-9) was purchased from Merck KGaA. Baicalin solution (15 mg/mL) was prepared by mixing baicalin into sterile saline. The mice were injected i.p. with 150 mg/kg BW baicalin once a day during hypoxic exposure.

### Measurement of right ventricular pressure.

The mice were anesthetized with an i.p. injection of pentobarbital sodium (50 mg/kg). The mice were orally intubated, and the lungs were ventilated using a mouse ventilator (Shinano Manufacturing Co., Ltd.). The ventilator settings were adjusted (tidal volume, 6 μL/g; frequency, 170–190/min). A 1.4F pressure–volume catheter (catalog SPR-671NR; Millar Instruments) was inserted into the right external jugular vein and advanced into the right ventricle to measure right ventricular pressure (RVP). RVP signals were relayed to pressure amplifiers (PCU-2000; AD Instruments) and then were continuously sampled using a PowerLab system (AD Instruments) and recorded on a computer using Chart software (AD Instruments). Heart rate was typically between 300 and 500 bpm under these conditions. If the heart rate fell below 300 bpm, the measurements were excluded from the analysis.

### Assessment of right ventricular hypertrophy.

The mice were euthanized by cervical dislocation, and their hearts were excised. The atria were removed, and the right ventricle (RV) was separated from the left ventricle (LV) and septum (S). The tissues were weighed, and RVH was calculated using Fulton’s index as the weight ratio of RV to LV plus intraventricular septum, that is, [RV/(LV + S)] (7).

### 2D histological analysis.

The mice were euthanized by cervical dislocation, and the left lungs were harvested for histological and IHC analyses. The pulmonary artery and trachea were perfused with 4% (wt/vol) PFA at constant pressures (100 cm H_2_O for the pulmonary artery and 25 cm H_2_O for the trachea) to fully distend the pulmonary blood vessels and airway, respectively. The excised lungs were fixed in 4% PFA overnight at 4°C, embedded in paraffin, and cut into 4 μm–thick sections. The Abs used for IHC staining included α-SMA (catalog C6198; Merck KGaA), CD31 (catalog DIA-310; Dianova), PGC-1α (catalog NBP1-04676; Novus Biologicals), and γH2.AX (catalog 9178; Cell Signaling Technology). Images were captured using an Olympus BX51 fluorescence microscope. Morphometry was performed on lung sections obtained from randomly chosen animals. Pulmonary remodeling was assessed using the percent wall thickness of parenchymal pulmonary arteries classified into small arteries (terminal bronchioles) and arterioles (acini or alveolar ducts). The percent SMC thickness was calculated as [(SMC thickness × 2)/vessel diameter] × 100. The pulmonary artery diameter was determined using the ImageJ software (NIH).

### CUBIC tissue-clearing protocol and 3D immunofluorescence staining.

CUBIC tissue clearing and 3D immunofluorescent staining were performed as previously described (12, 46). Mice were anesthetized with an i.p. injection of pentobarbital sodium (60 mg/kg). For transcardial perfusion, a 25-gauge needle was inserted into the LV through the apex. Mice were transcardially perfused with 150 mL of 4% (wt/vol) PFA in PBS containing 10 U/mL heparin and 20 mL of 50% (vol/vol) CUBIC-1 in sequence. The postcaval lobes of right lung were excised and continuously immersed in 30 mL of the CUBIC-1 reagent at 37°C with gentle shaking for 1 week for wholemount staining or for 1 day for samples in which ECs were labeled by tdTomato fluorescent proteins. The reagent was exchanged every day.

For wholemount immunostaining, the lungs were washed 3 times with PBS for 30 minutes each time at room temperature with gentle shaking after clearing out the CUBIC-1 reagent, immersed in 20% (wt/vol) sucrose in PBS at room temperature, and then frozen in OCT compound (Sakura Finetek) at –80°C overnight. The lungs were subjected to immunostaining with Cy3-conjugated anti–α-SMA Ab (C6198; Merck KGaA) and Hoechst 33342 (H3570; Thermo Fisher Scientific) in 2% (vol/vol) Triton X-100 for 3 days at 4°C with gentle shaking. The stained samples were then washed with 10 mL of PBS with 2% Tween 3 times for 30 minutes each time at 4°C with gentle shaking and immersed in CUBIC-2 reagent.

### 3D image acquisition.

3D image acquisition was performed as previously described (12). Images of tissue-cleared, immunofluorescence-labeled, or fluorescent protein–labeled samples were acquired using multiphoton excitation fluorescence microscopy SP8 (Leica Microsystems Ltd.) equipped with a ×5 (HCX PL Fluotar ×5/0.15, numerical aperture (NA) = 0.15, working distance (WD) = 13.7 mm), ×10 (HCX PL Fluotar ×10/0.30, NA = 0.3, WD = 11 mm), and ×20 (HCX APO ×20/0.75 immersion correction ring CS2, NA = 0.75, WD = 0.68 mm). The pixel size of each obtained image with ×5, ×10, and ×20 objective lens was *x* = 4.54, 2.27, and 1.13 μm and *y* = 4.54, 2.27, and 1.13 μm, respectively; *z*-step was set to 13.2, 6.6, and 2.2 μm, respectively. The samples were immersed in the CUBIC-2 reagent during image acquisition. The tissue was excited at wavelengths of 800 and 900 nm using a Ti:sapphire laser (Chameleon Vision II; Coherent). Image processing and 3D reconstruction (maximum intensity projection and volume rendering) were performed using Leica application suite X (Leica Microsystems Ltd.).

### 3D lineage-tracing experiment.

A 3D lineage-tracing experiment of ECs was performed to evaluate 3-dimensionally the proliferation of preexisting ECs during angiogenesis. *VE-cadherin-CreER^T2^*; *Rosa26-lsl-tdTomato* mice were injected i.p. with a single dose of tamoxifen (5 mg/kg BW) (Merck KGaA) dissolved in corn oil (Wako Pure Chemical Industries, Ltd.) at 5 weeks of age and started being housed in a hypoxic chamber or drug administration at 8 weeks of age. The lungs were collected at 11 weeks of age, and 3D images were acquired. The angiogenic response was quantitatively evaluated as the angiogenesis index, as previously described (12). The neovessel length and lung circumference were measured using ImageJ software (NIH).

### Quantification of vessel density.

We performed binary conversion of the acquired 3D images and calculated the integral of the area of interest to quantify vascular density in transparent lungs, as previously described (22).

### Image similarity analysis by feature matching.

We implemented the AKAZE algorithm (13), which is used for feature detection and description, in Python (47). Evaluating 3D images in their raw form as *x*–*y* plain images with *z*-stacks is difficult; therefore, we adopted a 2.5D approach, which uses 2D image representations of 3D objects (3D-reconstructed images) for feature matching, because shapes are more easily identified (48). Before extraction of feature points of the images, we performed grayscale conversion of 3D reconstructed images to improve the feature extraction accuracy, and the image size was uniformly converted to 256 × 256 pixels. The similarity between 2 images was evaluated by automatically extracting and matching image feature points using the AKAZE algorithm. The degree of similarity between the images was then calculated by comparing the similarity score, which was defined as the mean distance between feature points. A lower similarity score corresponded to more similar images.

### RNA isolation and qRT-PCR.

The mice were euthanized by cervical dislocation, and their lungs were excised. Total RNA from lung tissues was extracted using an RNeasy Mini Kit (Qiagen). Reverse transcription was performed with 1 μg of total RNA, random hexamers, and reverse transcriptase (ReverTraAce; TOYOBO). qRT-PCR was performed using FastStart Essential DNA Green Master (Roche) in a LightCycler 480 System II (Roche). The expression level of each gene was normalized to that of 18s rRNA. The sequences of the PCR primers are listed in Supplemental Table 1.

### Western blotting.

The mice were euthanized by cervical dislocation, and their lungs were excised. The extracted proteins from lung tissues were separated using SDS PAGE and electrophoretically transferred to PVDF membranes (Millipore). The primary Abs used included PGC-1α (catalog NBP1-04676; Novus Biologicals) and actin (catalog MA511869; Thermo Fisher Scientific). For the detection of protein carbonylation, Protein Carbonyl Western Blot Detection Kit (SHIMA Laboratories Co., Ltd) was used. Membranes were incubated with 2,4-dinitrophenylhydrazine solution to form the stable derivative of carbonyl groups. The derivatives were detected by anti-dinitrophenyl Ab. Membranes were exposed to HRP-conjugated secondary Abs, and signals were detected using the Pierce ECL Plus Western Blotting Substrate (Thermo Fisher Scientific). Densitometric analysis of proteins on Western blots was performed using ImageJ software (NIH) and normalized to the indicated internal control proteins.

### Data availability.

Underlying data for the manuscript can be accessed in the supplemental material.

### Statistics.

Data are expressed as mean ± SD. Statistical significance for comparison among means was evaluated using Pearson’s χ^2^ test, Student’s *t* test, or 1-way ANOVA followed by post hoc Tukey’s multiple-comparison tests. A *P* value < 0.05 was considered statistically significant. All statistical analyses were performed using Statistical Package for the Social Sciences, version 19 (IBM), and graphs were made using R, version 4.1.0 (R development Core Team). *P* values are indicated in individual figure legends.

### Study approval.

All animal experiments were approved by the Ethics Committee for Animal Experiments of the University of Tokyo and strictly adhered to the guidelines of the University of Tokyo for animal experiments.

## Author contributions

TF, NT, HH, M Hatano, and IK contributed to the study conception and design. TF, HH, SI, GN, H Tokiwa, MK, SM, TS, H Takiguchi, TY, and YK contributed to data acquisition. TF, NT, HH, SI, MK, and S Nishimura contributed to the data analysis. TF, NT, S Nomura, KU, M Harada, H Toko, ET, HA, and IK contributed to the data interpretation. TF, NT, HM, and IK drafted the manuscript. All authors participated in revising it critically for important intellectual content.

## Supplementary Material

Supplemental data

Supplemental video 1

Supplemental video 10

Supplemental video 11

Supplemental video 12

Supplemental video 2

Supplemental video 3

Supplemental video 4

Supplemental video 5

Supplemental video 6

Supplemental video 7

Supplemental video 8

Supplemental video 9

Supporting data values

## Version 1 09/08/2023

Electronic publication

## References

[1] Rich JD, Rich S (2014). Clinical diagnosis of pulmonary hypertension. Circulation.

[2] Tuder RM (2007). Pathology of pulmonary hypertension. Clin Chest Med.

[3] Tuder RM (2001). Expression of angiogenesis-related molecules in plexiform lesions in severe pulmonary hypertension: evidence for a process of disordered angiogenesis. J Pathol.

[4] Tuder RM (1995). Increased gene expression for VEGF and the VEGF receptors KDR/Flk and Flt in lungs exposed to acute or to chronic hypoxia. Modulation of gene expression by nitric oxide. J Clin Invest.

[5] Cho YJ (2009). Temporal changes of angiopoietins and Tie2 expression in rat lungs after monocrotaline-induced pulmonary hypertension. Comp Med.

[6] Yu AY (1999). Impaired physiological responses to chronic hypoxia in mice partially deficient for hypoxia-inducible factor 1alpha. J Clin Invest.

[7] Brusselmans K (2003). Heterozygous deficiency of hypoxia-inducible factor-2alpha protects mice against pulmonary hypertension and right ventricular dysfunction during prolonged hypoxia. J Clin Invest.

[8] Partovian C (2000). Adenovirus-mediated lung vascular endothelial growth factor overexpression protects against hypoxic pulmonary hypertension in rats. Am J Respir Cell Mol Biol.

[9] Abe K (2010). Formation of plexiform lesions in experimental severe pulmonary arterial hypertension. Circulation.

[10] Sitapara R (2021). SU5416 plus hypoxia but not selective VEGFR2 inhibition with cabozantinib plus hypoxia induces pulmonary hypertension in rats: potential role of BMPR2 signaling. Pulm Circ.

[11] Susaki EA (2014). Whole-brain imaging with single-cell resolution using chemical cocktails and computational analysis. Cell.

[12] Fujiwara T (2021). Three-dimensional visualization of hypoxia-induced pulmonary vascular remodeling in mice. Circulation.

[13] Zhang J (2018). An improved randomized local binary features for keypoints recognition. Sensors (Basel).

[14] Rabinovitch M (2008). Molecular pathogenesis of pulmonary arterial hypertension. J Clin Invest.

[15] Arany Z (2008). HIF-independent regulation of VEGF and angiogenesis by the transcriptional coactivator PGC-1alpha. Nature.

[16] Saint-Geniez M (2013). PGC-1α regulates normal and pathological angiogenesis in the retina. Am J Pathol.

[17] Ryan JJ (2013). PGC1α-mediated mitofusin-2 deficiency in female rats and humans with pulmonary arterial hypertension. Am J Respir Crit Care Med.

[18] Ye JX (2016). Suppression of endothelial PGC-1α is associated with hypoxia-induced endothelial dysfunction and provides a new therapeutic target in pulmonary arterial hypertension. Am J Physiol Lung Cell Mol Physiol.

[19] Hong KH (2008). Genetic ablation of the BMPR2 gene in pulmonary endothelium is sufficient to predispose to pulmonary arterial hypertension. Circulation.

[20] Zhang K (2011). Baicalin increases VEGF expression and angiogenesis by activating the ERRα/PGC-1α pathway. Cardiovasc Res.

[21] Huang S (2014). Baicalin attenuates transforming growth factor-β1-induced human pulmonary artery smooth muscle cell proliferation and phenotypic switch by inhibiting hypoxia inducible factor-1α and aryl hydrocarbon receptor expression. J Pharm Pharmacol.

[22] Hasegawa S (2019). Comprehensive three-dimensional analysis (CUBIC-kidney) visualizes abnormal renal sympathetic nerves after ischemia/reperfusion injury. Kidney Int.

[23] Thenappan T (2018). Pulmonary arterial hypertension: pathogenesis and clinical management. BMJ.

[24] Masaki T (2021). Aryl hydrocarbon receptor is essential for the pathogenesis of pulmonary arterial hypertension. Proc Natl Acad Sci U S A.

[25] Rius-Perez S (2020). PGC-1α, inflammation, and oxidative stress: an integrative view in metabolism. Oxid Med Cell Longev.

[26] Valle I (2005). PGC-1alpha regulates the mitochondrial antioxidant defense system in vascular endothelial cells. Cardiovasc Res.

[27] Schulz E (2008). Suppression of the JNK pathway by induction of a metabolic stress response prevents vascular injury and dysfunction. Circulation.

[28] Xiong S (2013). Peroxisome proliferator-activated receptor γ coactivator-1α is a central negative regulator of vascular senescence. Arterioscler Thromb Vasc Biol.

[29] Culley MK (2021). Frataxin deficiency promotes endothelial senescence in pulmonary hypertension. J Clin Invest.

[30] van der Feen DE (2020). Cellular senescence impairs the reversibility of pulmonary arterial hypertension. Sci Transl Med.

[31] Handschin C (2003). An autoregulatory loop controls peroxisome proliferator-activated receptor gamma coactivator 1alpha expression in muscle. Proc Natl Acad Sci U S A.

[32] Czubryt MP (2003). Regulation of peroxisome proliferator-activated receptor gamma coactivator 1 alpha (PGC-1 alpha) and mitochondrial function by MEF2 and HDAC5. Proc Natl Acad Sci U S A.

[33] Southgate RJ (2005). PGC-1alpha gene expression is down-regulated by Akt- mediated phosphorylation and nuclear exclusion of FoxO1 in insulin-stimulated skeletal muscle. FASEB J.

[34] Canto C (2009). AMPK regulates energy expenditure by modulating NAD+ metabolism and SIRT1 activity. Nature.

[35] Jager S (2007). AMP-activated protein kinase (AMPK) action in skeletal muscle via direct phosphorylation of PGC-1alpha. Proc Natl Acad Sci U S A.

[36] Omura J (2016). Protective roles of endothelial AMP-activated protein kinase against hypoxia-induced pulmonary hypertension in mice. Circ Res.

[37] Garat CV (2020). CREB depletion in smooth muscle cells promotes medial thickening, adventitial fibrosis and elicits pulmonary hypertension. Pulm Circ.

[38] Savai R (2014). Pro-proliferative and inflammatory signaling converge on FoxO1 transcription factor in pulmonary hypertension. Nat Med.

[39] Sofer A (2018). Therapeutic engagement of the histone deacetylase IIA-myocyte enhancer factor 2 axis improves experimental pulmonary hypertension. Am J Respir Crit Care Med.

[40] Zurlo G (2018). Sirtuin 1 regulates pulmonary artery smooth muscle cell proliferation: role in pulmonary arterial hypertension. J Hypertens.

[41] Feng W (2019). Paclitaxel alleviates monocrotaline-induced pulmonary arterial hypertension via inhibition of FoxO1-mediated autophagy. Naunyn Schmiedebergs Arch Pharmacol.

[42] Okabe K (2014). Neurons limit angiogenesis by titrating VEGF in retina. Cell.

[43] Madisen L (2010). A robust and high-throughput Cre reporting and characterization system for the whole mouse brain. Nat Neurosci.

[44] Asikainen TM (2005). Stimulation of HIF-1alpha, HIF-2alpha, and VEGF by prolyl 4-hydroxylase inhibition in human lung endothelial and epithelial cells. Free Radic Biol Med.

[45] Ciuclan L (2011). A novel murine model of severe pulmonary arterial hypertension. Am J Respir Crit Care Med.

[46] Tainaka K (2014). Whole-body imaging with single-cell resolution by tissue decolorization. Cell.

[48] Nakao T (2018). Deep neural network-based computer-assisted detection of cerebral aneurysms in MR angiography. J Magn Reson Imaging.

