# Supplementary material for: PGC-1α–mediated angiogenesis prevents pulmonary hypertension in mice
Source: JCI Insight. 2023 Sep 8;8(17):e162632. doi: 10.1172/jci.insight.162632 (PMC10544206; doi:10.1172/jci.insight.162632)
Supplement: Supplemental data [file jciinsight-8-162632-s082.pdf]

Supplemental Materials

Title: PGC-1α-mediated angiogenesis prevents pulmonary hypertension in mice

Brief title: PGC-1α-mediated angiogenesis in pulmonary hypertension

Takayuki Fujiwara[1,2,7], Norifumi Takeda, MD[1,#], Hironori Hara[1,3], Satoshi Ishii[1], Genri Numata[1,3], Hiroyuki Tokiwa[1], Manami Katoh[1], Sonoko Maemura[1], Takaaki Suzuki[1], Hiroshi Takiguchi[1], Tomonobu Yanase[1], Yoshiaki Kubota[6], Seitaro Nomura[1,4], Masaru Hatano[1], Kazutaka Ueda[1], Mutsuo Harada[1,5], Haruhiro Toko[1], Eiki Takimoto[1], Hiroshi Akazawa[1], Hiroyuki Morita[1], Satoshi Nishimura[7] and Issei Komuro[1,#]

[1]Department of Cardiovascular Medicine, The University of Tokyo Hospital, 7-3-1 Hongo, Bunkyo-ku, Tokyo 113-8655, Japan
[2]Department of Computational Diagnostic Radiology and Preventive Medicine,
[3]Department of Advanced Translational Research and Medicine in Management of Pulmonary Hypertension
[4]Department of Therapeutic Strategy for Heart Failure,
[5]Department of Advanced Clinical Science and Therapeutics, Graduate School of Medicine, The University of Tokyo, 7-3-1 Hongo, Bunkyo-ku, Tokyo 113-8655, Japan
[6]Department of Anatomy, Keio University School of Medicine, 35 Shinanomachi, Shinjuku-ku, Tokyo 160-8582, Japan
[7]Center for Molecular Medicine, Jichi Medical University, 3311-1 Yakushiji, Shimotsuke, Tochigi 329-0498, Japan

Address for correspondence:
Issei Komuro
Department of Cardiovascular Medicine
The University of Tokyo Hospital
7-3-1 Hongo, Bunkyo-ku, Tokyo 113-8655, Japan
Phone: +81-3-3815-5411
Fax: +81-3-5800-2087
Email: komuro_tky2000@yahoo.co.jp

Norifumi Takeda
Department of Cardiovascular Medicine
The University of Tokyo Hospital
7-3-1 Hongo, Bunkyo-ku, Tokyo 113-8655, Japan
Phone: +81-3-3815-5411
Fax: +81-3-5800-9182
Email: norifutakeda@gmail.com

Supplemental Table S1

List of primer sequences used for qRT-PCR analysis

Genes	Forward primer	Reverse primer
18s	CGGCTACCACATCCAAGGAA	AGCTGGAATTACCGCGGC
Vegfa	ATCTTCAAGCCGTCCTGTGT	GCATTCACATCTGCTGTGCT
Ppargc1a	CCGAGAATTCATGGAGCAAT	TTTCTGTGGGTTTGGTGTGA
Cdkn1a	CACAGCTCAGTGGACTGGAA	ACCCTAGACCCACAATGCAG
Tnf	TAGCCAGGAGGGAGAACAGA	TTTTCTGGAGGGAGATGTGG
Il6	GATGATGCACTTGCAGAAAACA	GGTACTCCAGAAGACCAGAGGAA

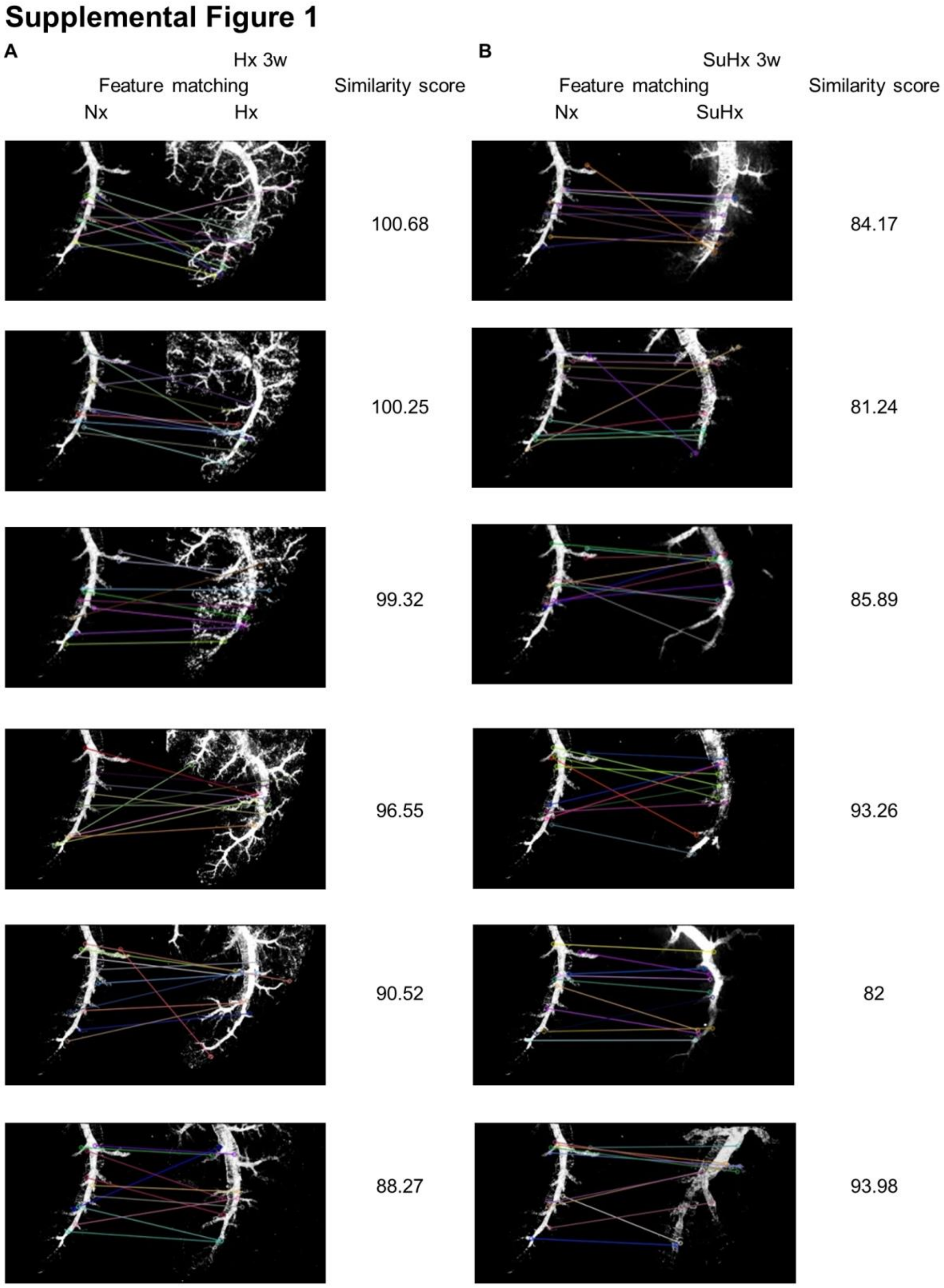

Supplemental figure 1. Feature matching and similarity score against 3D reconstructed SMC image of normoxia in multiple PH models

All set of comparison images used to calculate the similarity score (Figure 1H) are shown. Image feature points from two reconstructed images depicting SMC remodeling were automatically extracted by AKAZE feature detector and descriptor, and the similarity score was calculated by mean distance between the matched feature points. Comparison images between normoxia (Nx) and hypoxia (Hx 3w, **A**) and SU5416/hypoxia mice (SuHx 3w, **B**).

Supplemental Figure 2

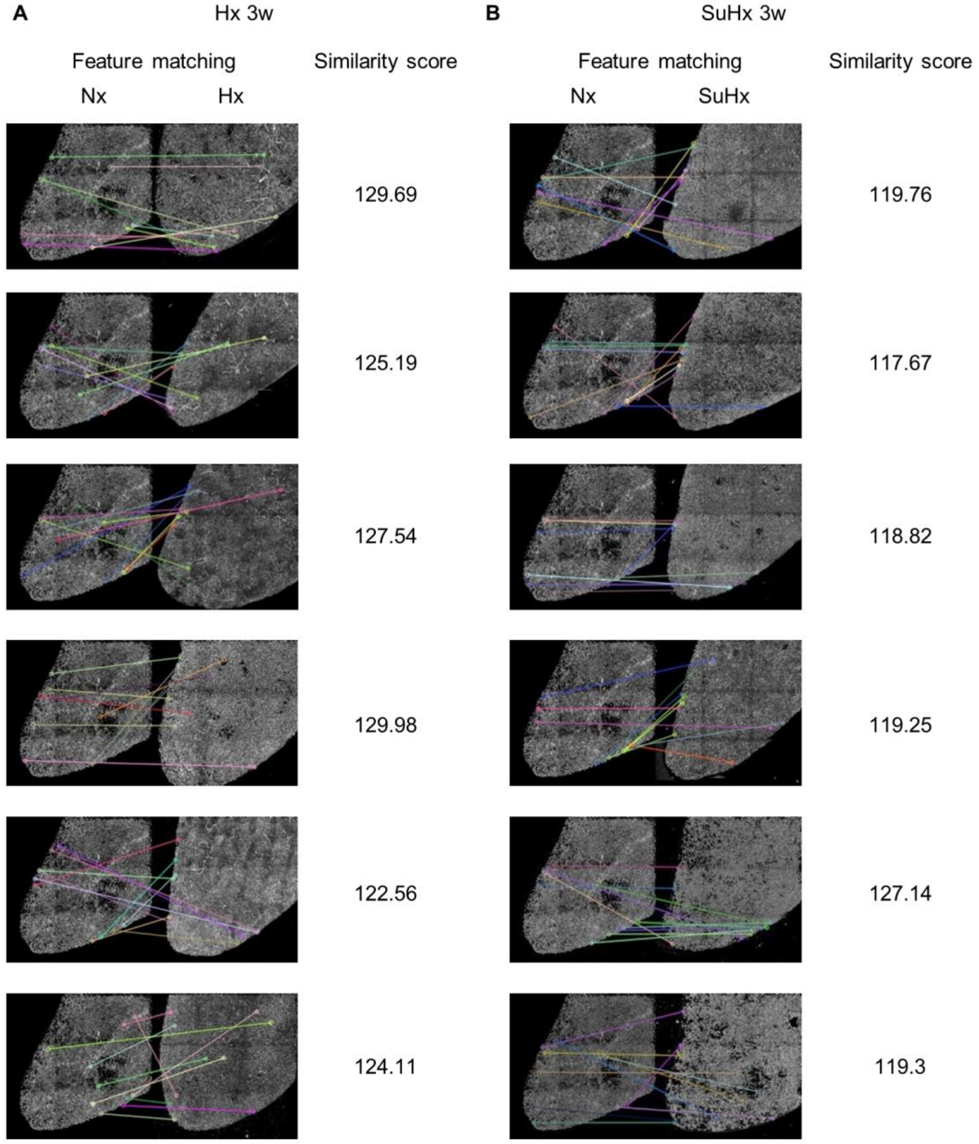

Supplemental figure 2. Feature matching and similarity score against 3D reconstructed EC lineage-tracing image of normoxia in multiple PH models

All set of comparison images used to calculate the similarity score (Figure 2C) are shown. Image feature points from two reconstructed images depicting EC lineage-tracing (angiogenesis) were automatically extracted by AKAZE feature detector and descriptor, and the similarity score was calculated by mean distance between the matched feature points. Comparison images between normoxia (Nx) and hypoxia (Hx 3w, **A**), SU5416/hypoxia mice (SuHx 3w, **B**).

Supplemental Figure 3

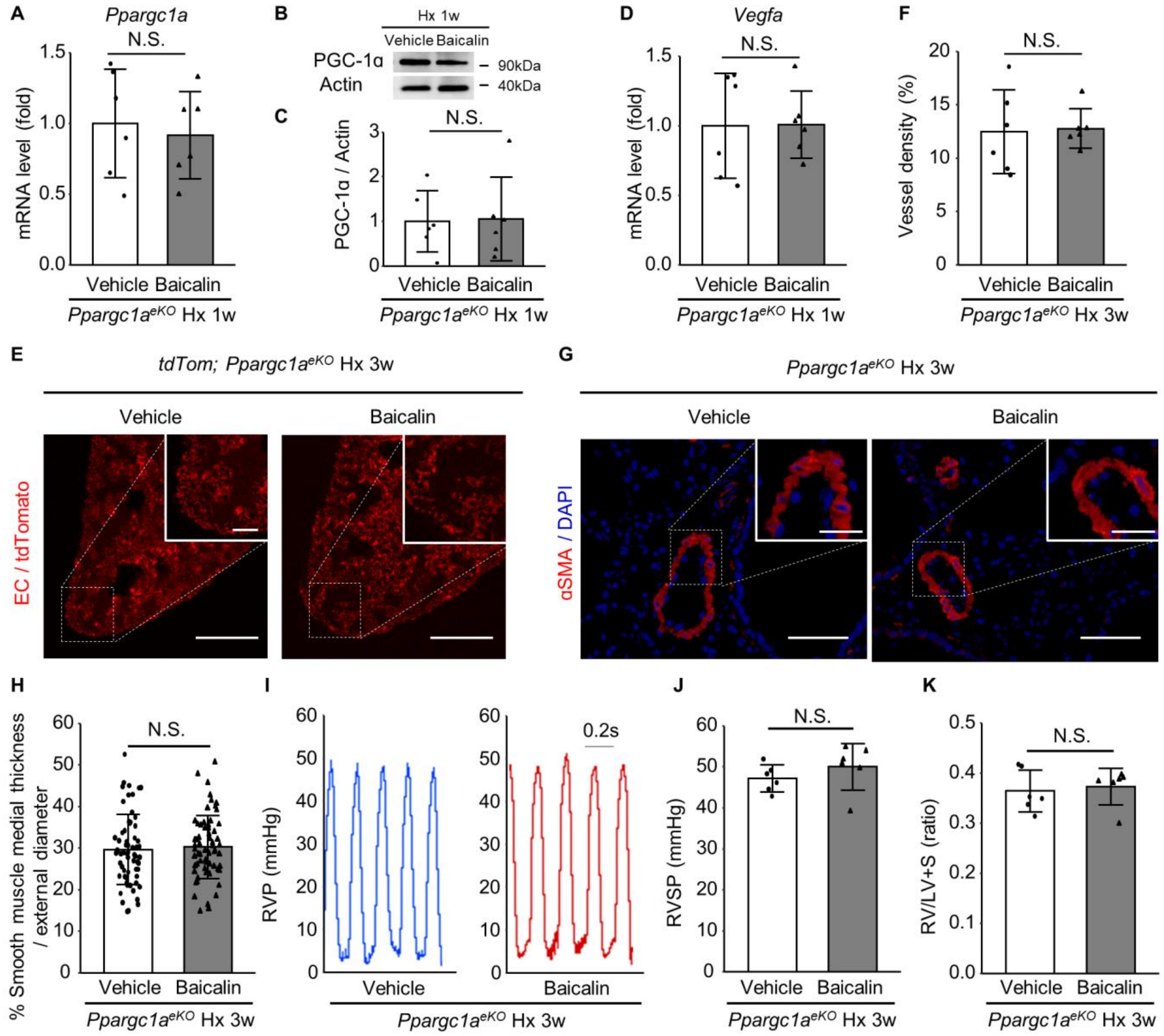

Supplemental Figure 3. Baicalin treatment did not enhance PGC-1α-mediated angiogenic response, nor attenuate Hx-PH in *Ppargc1a*^{eKO} mice

Ppargc1a^{eKO} mice were treated with baicalin (150 mg/kg/day) or vehicle during 3 weeks of hypoxic conditions. **(A)** Relative expression of *Ppargc1a* measured one week after hypoxic exposure (n = 6 per group). **(B, C)** Western blot analysis of PGC-1α protein in the lung. One representative blot **(B)** and quantified PGC-1α levels **(C)** are presented (n = 6 mice per group). **(D)** Relative expression of *Vegfa* one week after hypoxic exposure (n = 6 mice per group). **(E, F)** Pulmonary ECs of *Ppargc1α*^{eKO} mice (*L1-Cre*; *Ppargc1α*$^{fl/fl}$) were labeled with tdTomato fluorescence by generating *L1-Cre*; *Rosa26-lsl-tdTomato*; *Ppargc1α*$^{fl/fl}$ (*tdTom*; *Ppargc1α*^{eKO}). The mice were exposed to Hx with administration of baicalin or vehicle for 3 weeks (Hx 3w). **(E)** Representative multiphoton images of the lung ECs. Scale bar: 500 µm (and 200 µm for the inset). **(F)** Quantification of EC volume density (n = 6 mice per group). **(G)** Representative 2D images showing αSMA-stained arterioles. Scale bar: 50 µm (and 20 µm for the inset). **(H)** Quantification of SMC thickness (n = 62 vessels from N = 4 mice per group). **(I)** Representative RVP data. **(J)** RVSP (n = 6 mice per group). **(K)** RVH (n = 6 mice per group). Data are representative of three independent experiments. Statistical significance for comparison between means was evaluated using a Student's t-test.

Supplemental Figure 4

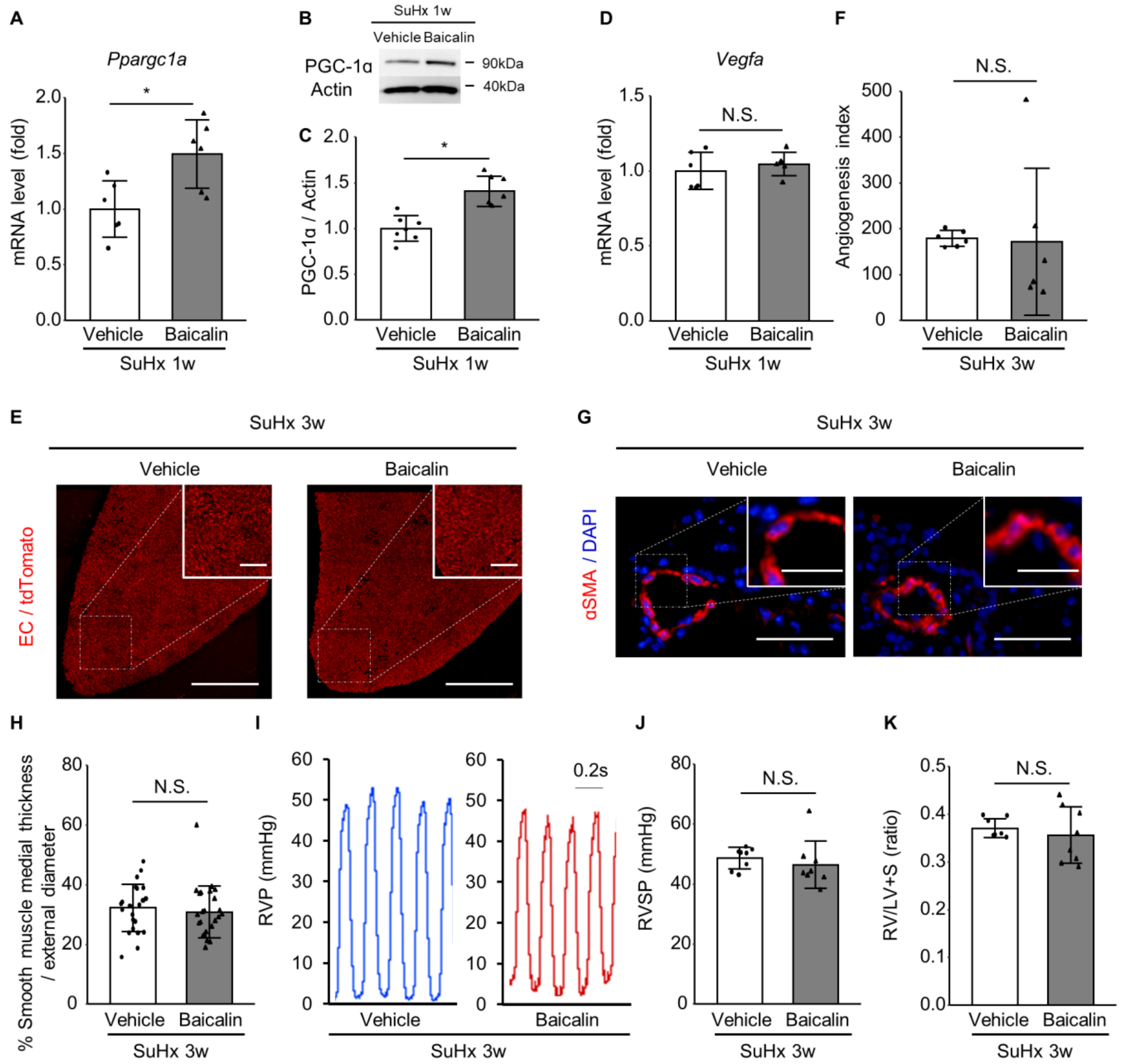

Supplemental Figure 4. Baicalin treatment increased PGC-1α expression in SuHx mice, but did not enhance angiogenic response or attenuate PH

SuHx mice were treated with baicalin (150 mg/kg/day) or vehicle during 3 weeks of hypoxic conditions. **(A)** Relative expression of *Ppargc1a* measured one week after hypoxic exposure (n = 6 per group). **(B, C)** Western blot analysis of PGC-1α protein in the lung. One representative blot **(B)** and quantified PGC-1α levels **(C)** are presented (n = 7 mice per group). **(D)** Relative expression of *Vegfa* one week after hypoxic exposure (n = 6 mice per group). **(E, F)** 3D EC linage-tracing experiment. **(E)** Representative 3D EC lineage-tracing images. Scale bar: 500 μm (and 100 μm for the inset). **(F)** Angiogenesis index (n = 6 mice per group). **(G)** Representative 2D images showing αSMA-stained arterioles. Scale bar: 50 μm (and 20 μm for the inset). **(H)** Quantification of SMC thickness (n = 24 vessels from N = 4 mice per group). **(I)** Representative RVP data. **(J)** RVSP (n = 8 mice per group). **(K)** RVH (n = 8 mice per group). Data are representative of three independent experiments. Statistical significance for comparison between means was evaluated using a Student's *t*-test. *$P < 0.05$.

Supplemental video legends

Supplemental video 1. 3D morphological analysis of pulmonary smooth muscle cells in mouse under normoxia

Wild-type mouse was housed in the normoxic chamber for control until the age of 11 weeks. SMCs stained with Cy3-conjugated anti-αSMA antibody were three-dimensionally shown. Nuclei were counterstained with Hoechst 33342.

Supplemental video 2. 3D morphological analysis of pulmonary smooth muscle cells in hypoxia (Hx) -induced pulmonary hypertension mouse model

Wild-type mouse was exposed to hypoxia for 3 weeks from the age of 8 weeks (Hx 3w). SMCs stained with Cy3-conjugated anti-αSMA antibody were three-dimensionally shown. Note the marked SMC elongation into the peripheral lung and/or hyperplasia-like structure. Nuclei were counterstained with Hoechst 33342.

Supplemental video 3. 3D morphological analysis of pulmonary smooth muscle cells in SU5416/hypoxia (SuHx) -induced PH mouse model

Wild-type mouse was subjected to hypoxia and treated with SU5416 for 3 weeks from the age of 8 weeks (SuHx 3w). SMCs stained with Cy3-conjugated anti-αSMA antibody were three-dimensionally shown. Note that SMC elongation into the peripheral lung observed in Hx-PH (Supplemental video 2) was less often in SuHx severe PH model. Nuclei were counterstained with Hoechst 33342.

Supplemental video 4. 3D lineage-tracing analysis of pulmonary endothelial cells in mouse under normoxia

VE-cadherin-CreER^{T2}; *Rosa26-lsl-tdTomato* mouse was treated with a single dose of tamoxifen at 5 weeks of age, leading to expression of tdTomato fluorescent protein in *VE-cadherin*-expressing ECs, and housed in the normoxic chamber for control until the age of 11 weeks. tdTomato-labeled cells were three-dimensionally shown.

Supplemental video 5. 3D lineage-tracing analysis of pulmonary endothelial cells in Hx-PH mouse model

VE-cadherin-CreER^{T2}; *Rosa26-lsl-tdTomato* mouse was treated with a single dose of tamoxifen at 5 weeks of age and exposed to hypoxia for 3 weeks from the age of 8 weeks (Hx 3w). tdTomato-labeled cells were three-dimensionally shown. Sprouting and elongation of the tdTomato-labeled pre-existing ECs, indicating angiogenesis, was evident in Hx 3w mild PH model.

Supplemental video 6. 3D lineage-tracing analysis of pulmonary endothelial cells in SuHx-PH mouse model

VE-cadherin-CreER^{T2}; *Rosa26-lsl-tdTomato* mouse was treated with a single dose of tamoxifen at 5 weeks of age, and subjected to hypoxia and treated with SU5416 for 3 weeks from the age of 8 weeks (SuHx 3w). tdTomato-labeled cells were three-dimensionally shown, and tdTomato-labeled neovessel formation from pre-existing ECs observed in Hx-PH (Supplemental video 5) was less often in SuHx 3w severe PH model.

Supplemental video 7. 3D lineage-tracing analysis of pulmonary endothelial cells in Hx-PH mouse model

VE-cadherin-CreER^{T2}; *Rosa26-lsl-tdTomato* mouse was treated with a single dose of tamoxifen at 5 weeks of age and exposed to hypoxia for 3 weeks from the age of 8 weeks (Hx

3w). tdTomato-labeled cells were three-dimensionally shown. tdTomato-labeled neovessel formation from pre-existing ECs was evident in Hx 3w mild PH model.

Supplemental video 8. 3D lineage-tracing analysis of pulmonary endothelial cells in CabHx-PH mouse model
VE-cadherin-CreER^{T2}; *Rosa26-lsl-tdTomato* mouse was treated with a single dose of tamoxifen at 5 weeks of age, and subjected to hypoxia and treated with cabozantinib for 3 weeks from the age of 8 weeks (SuHx 3w). tdTomato-labeled cells were three-dimensionally shown, and tdTomato-labeled neovessel formation from pre-existing ECs was less obvious in CabHx-PH mice compared to Hx-PH mice (Supplemental video 7).

Supplemental video 9. 3D lineage-tracing analysis of pulmonary endothelial cells in Hx-PH mouse model with vehicle
VE-cadherin-CreER^{T2}; *Rosa26-lsl-tdTomato* mouse was treated with a single dose of tamoxifen at 5 weeks of age, and subjected to hypoxia and treated with saline (vehicle) for 3 weeks from the age of 8 weeks. tdTomato-labeled pre-existing ECs and derivatives, indicating angiogenesis, were three-dimensionally shown.

Supplemental video 10. 3D lineage-tracing analysis of pulmonary endothelial cells in Hx-PH mouse treated with baicalin, a flavonoid having a PGC-1α activation effect
VE-cadherin-CreER^{T2}; *Rosa26-lsl-tdTomato* mouse was treated with a single dose of tamoxifen at 5 weeks of age, and subjected to hypoxia and treated with baicalin for 3 weeks from the age of 8 weeks. tdTomato-labeled pre-existing ECs were three-dimensionally shown. Note that sprouting and elongation of the tdTomato-labeled pre-existing ECs, indicating angiogenesis, was enhanced by baicalin treatment, compared to vehicle control (Supplemental video 9).

Supplemental Video 11. 3D lineage-tracing analysis of pulmonary endothelial cells in SuHx-PH mouse model with vehicle
VE-cadherin-CreER^{T2}; *Rosa26-lsl-tdTomato* mouse was treated with a single dose of tamoxifen at 5 weeks of age, and subjected to hypoxia and treated with SU5416 and saline (vehicle) for 3 weeks from the age of 8 weeks. tdTomato-labeled cells were three-dimensionally shown.

Supplemental Video 12. 3D lineage-tracing analysis of pulmonary endothelial cells in SuHx-PH mouse model with baicalin
VE-cadherin-CreER^{T2}; *Rosa26-lsl-tdTomato* mouse was treated with a single dose of tamoxifen at 5 weeks of age, and subjected to hypoxia and treated with SU5416 and baicalin for 3 weeks from the age of 8 weeks. Neovessel formation from pre-existing ECs was not evident as well as SuHx-PH mouse with vehicle (Supplemental Video 11)
